# Examining the association between work stress, life stress and obesity among working adult population in Canada: findings from a nationally representative data

**DOI:** 10.1186/s13690-022-00865-8

**Published:** 2022-03-29

**Authors:** Nigatu Regassa Geda, Cindy Xin Feng, Yamei Yu

**Affiliations:** 1 Center for Population Studies, College of Development Studies, Addis Ababa University, Addis Ababa, Ethiopia; 2 Department of Community Health and Epidemiology, Faculty of Medicine, Dalhousie University, Halifax, Nova Scotia Canada; 3 School of Epidemiology and Public Health, Faculty of Medicine, University of Ottawa, Ottawa, Canada

**Keywords:** Obesity, Work stress, Life stress, Lifestyle, Random effect

## Abstract

**Background:**

Obesity is a priority public health concern in Canada and other parts of the world. The study primarily aims at assessing the role of  self-perceived work and life stress on obesity among working adults in Canada.

**Methods:**

The study was conducted based on a total of 104,636 Canadian adults aged 18 and above, extracted from the 2017–2018 Canadian Community Health Survey (CCHS) data. We used a mixed-effect logistic regression model to determine the possible association between two stress variables and obesity, controlling for other variables in the model. The random effect term accounts for the correlation among the observations from the same health region.

**Results:**

A total of 63,815 adult respondents (aged 18 and above) who were working during the 12 months prior to the survey were studied. Of those, 18.7% were obese based on their self-reported BMI > =30.0 kg/m^2^. More than two-thirds of the respondents reported that their stress level is a bit stressful to extremely stressful. The results of multivariable mixed-effect logistic regression showed that the odds of obesity were 1.432 times (95% CI: 1.248–1.644) among those who reported extremely work-related stress, compared to those who had no work-related stress. Perceived life stress was not significantly associated with obesity risk among working adult population, after adjusting other factors.

**Conclusion:**

The study concluded that obesity among Canadian adults is 18.7% of the working adult population being obese. Given the reported high prevalence of stress and its effect on obesity, the findings suggested improving social support systems, individual/group counseling, and health education focusing on work environments to prevent and manage stressors and drivers to make significant program impacts.

**Supplementary Information:**

The online version contains supplementary material available at 10.1186/s13690-022-00865-8.

## Background

Obesity is becoming an increasingly important public health concern around the globe, including in the developing world [1, 2]. Obesity is associated with an increased risk of many chronic conditions, such as cancer, hypertension, and type 2 diabetes [3]. It is also exerting adverse impacts on quality of life and increases the risk of premature death [4, 5]. In Canada, obesity contributes 61–74% of type 2 diabetes cases and about 20% of premature deaths among adults [5]. The economic cost of obesity in Canada is also huge. Tran and colleagues (2013) estimated that aggregated annual costs of obesity in Canada ranged from 1.27 to 11.08 billion dollars, of which direct costs accounted for 37.2 to 54.5% of total annual costs [6]. Similarly, most recent data indicate that Canada spends roughly 11% of its total health expenditure on treating medical conditions that arise as complications of obesity [7].

Given these serious consequences for obesity, understanding the etiology of obesity by identifying its important predictors is essential to help health care policymakers to develop prevention and intervention strategies for reducing obesity. Obesity is a complex phenomenon as several biological, behavioral, societal factors and pathways are involved [8, 9]. The social-ecological model (SEM) (also known as the ecological model) could be a plausible theoretical model to explain obesity in most societies. A fundamental strength of all SEMs is that their focus on multiple levels of influence expands the range of determinants considered and broadens options for interventions [10–12]. Interventions that target multiple levels of influence are expected to reach a greater proportion of the population and establish settings that result in sustainable behavior changes [12]. SEMs generally include considerations of individual (e.g., personal beliefs, behaviors, and motivations), interpersonal (e.g., family, friends, neighborhood, segregation and social networks, social interaction, and peers), physical environment (e.g., schools, home, and work sites), and macro-level (e.g., health policy, food marketing, and social norms) variables [13]. The SEM offers a framework to analyze the contextual variables influencing eating behavior, stress, sedentary lifestyles, and other associated factors, which requires a multilevel statistical analysis. In addition, the SEM can guide research and planning of comprehensive diet-related behavior interventions [12, 14]. In our study, we particularly focus on the predictors at the individual and cluster (i.e., health regions) levels, given the availability of the information collected in the current study.

Of the individual-level characteristics, the socioeconomic variables, including gender, ethnicity, educational status, income, and living arrangement, were reported to be associated with obesity [15–17]. The inverse association between obesity and leisure-time physical activity is well documented [1, 15, 18]. Sedentary behaviors (such as time spent watching television or videos or using a computer, reading, sitting) are key predictors of obesity in Canada [19]. Of the neighborhood-level factors, obesity was associated with neighborhood physical and sociocultural characteristics, such as neighborhood built environment characteristics (e.g., land use mix, population density, street connectivity, access to recreational facilities, a higher density of fast-food restaurants and more [19, 20]. In a recent report, obesity was also found to be more prevalent in the most socioeconomically deprived areas than in the least deprived. In Halifax, for example, 25.5% of people in the lowest SES areas were obese compared with 11.2% of people in the highest SES areas [19]. For the participants who live close to each other, their corresponding environmental characteristics would tend to be more similar. As a result, relationships between the neighborhood environment and obesity are clearly embedded in a geographical context. However, many neighborhood environment characteristics are not collected in the data. Multilevel modeling allows us to explore the importance of social context by dividing the total variation into individuals and clusters to be assessed separately. Therefore, in this study, a mixed effect logistic regression including health region as a random effect term is considered to give an enriched picture of the complexity of obesity.

In the very fast-changing social and economic life, the effects of life and work stress have been less emphasized in the study of obesity. Stress, a function of biological and behavioral effects [21], stimulates increased body hormones called ‘cortisol’ [22–24]. The increase in such hormones leads to craving unhealthy food and causing to eat more than one normally would [22]. Therefore, a better understanding of the role of stress in obesity will help us develop effective treatment strategies in daily clinical practice. The very few available studies focus on a specific population group such as Canadian adolescents [17, 25] or sex-specific estimation of prevalence [1] or considered very few exposures and control variables to measure the effects [26, 27]. Filling this empirical gap is useful for the Canadian health research landscape, given stress is a lifestyle variable that could be potentially modified.

Therefore, the primary objective of the present study is to assess the role of self-reported work and life stress in obesity among the Canadian working population based on a multilevel mixed effect model using the Canadian Community Health Survey, a recent nationally representative data of Canada collected in the year 2017 by Statistics Canada.

## Methods

### Data source, study design and study population

The study is based on data from the 2017–2018 Canadian Community Health Survey (CCHS), which is a multistage complex cross-sectional survey that collected socioeconomic and health information from 113,290 participants aged 12+ drawn from all Canadian provinces and territories [28]. For the present analysis, data for a total of 63,815 adult respondents (aged 18 and above) who were working during the 12 months prior to the survey were extracted. The detailed description of methods, design, instruments, participants, and sampling frame has previously been published by Statistics Canada: http://www23.statcan.gc.ca/imdb/p2SV.pl?Function=getSurvey&SDDS=3226.

### Study variables

#### Dependent variable

Body Mass Index (BMI) was defined as the ratio of self-reported weight (kg) and the square of self-reported height (m). In CCHS, participants are categorized into obese (BMI > =30.0 kg/m^2^), overweight (BMI 25.0–29.9 kg/m^2^), normal (18.5–24.9 kg/m^2^) and underweight < 18.5 kg/m2) [29, 30]. The outcome variable was coded as “0” for non-obese (BMI < 30 kg/m^2^) and “1” if the respondent reported BMI > =30 kg/m^2^ [30].

#### Primary explanatory variables of interest

The main explanatory variables in this study are the levels of work and life stress respondents experienced for 12 months preceding the survey. The question about the work stress question was asked as ‘what would you say most days at work were?’. The question about life stress was `thinking about the amount of stress in your life, what would you say most days are?'. Both stress measures were self-reported exposures, and participants’ responses were coded into five categories: not at all stressful, not very stressful, a bit stressful, quite a bit stressful, and extremely stressful. A bit stressful stands for mild stress, quite a bit stressful and extremely stressful represent moderate and severe stress levels in an ordinary language.

#### Other explanatory variables

The analysis included a number of control variables such as age, sex, household income, educational status, ethnicity, marital status, household food security status, number of working hours per week and type of employment. Age was classified into five groups (years):18–24, 25–34, 35–50, 51–64 and 65 and above. Ethnicity was grouped as white, aboriginal, or visible minority. The CCHS defined visible minorities per the Employment Equity Act of Canada as “persons, other than Aboriginal peoples, who are non-Caucasian in race or non-white in color” [31]. Respondents’ educational status was categorized into three groups: less than secondary school graduation, secondary school graduation, and post-secondary certificate diploma/university education. Marital status was defined as: married, common-law, widowed/divorced/separated, and single/never married. Respondents were grouped into five household income categories based on Canadian National average household income reported in past income tax reporting year, with $20,000 increments beginning with zero. It was categorized as no income or less than $20,000, $20,000 to $39,999, $40,000 to $59,999, $60,000 to $79,999, and $80,000 or more per year. Household food access was categorized into food secured, moderately food insecure and severely food insecure. The Household Food Security Survey Module in the CCHS is an 18–question, standardized and validated scale of food insecurity severity that measures inadequate or insecure access to food due to financial constraints [32]. Type of employment was categorized as employee and self-employed. Physical activity was classified according to World Health Organization (WHO) recommendations (active, moderately active, mildly active, sedentary) [33]. The measure was developed based on a list of moderate physical activities with the corresponding minimum amount of time required for a healthy life [33].

### Statistical analyses

Characteristics of the study participants were presented by the status of obesity. The respondents within the same health region/cluster may have similarities in the likelihood of the outcome [34]. Therefore, mixed-effects models were employed in the current study with a random intercept term incorporated in the model to account for the correlation among the observations from the same health region. Multicollinearity among the explanatory variables was checked using the Variance Inflation Factor (VIF) [35]. Covariates with VIF > 2.5 are considered to have multicollinearity. The analysis began with bivariate mixed-effects logistic regression analyses to examine the associations between each explanatory variable and the outcome variable. As a rule of thumb, potential variables with a *p* < 0.20 were selected for the multivariable mixed-effect logistic regression [35]. The manual backward selection was used to develop the main effects model, retaining only variables with *p* < 0.05. Two-way interactions of work and life stress variables (main exposure variables of interest) with other explanatory variables were assessed by entering the product of two hypothesized variables.

The intra-class correlation coefficient (ICC) is often used in multilevel analysis, which is calculated as the amount of between-cluster variance to the sum of between- and within-cluster error variances. As a rule of thumb, ICC close to zero indicates perfect independence of residuals, i.e., observations do not depend on the clusters considered. A high ICC close to 1 indicates high similarity between values from the same group. Values of ICC of less than 0.50, between 0.50 and 0.75, between 0.76 and 0.90 and greater than 0.90 indicate low, moderate, high and very high correlations, respectively [36]. ICC was computed based on the estimated variance of the final multivariable model [37, 38]. For modeling dichotomous clustered outcomes, scales between cluster-level and individual-level error variances are different. As a result, explaining the partitioning of total variance and ICC for dichotomous outcomes is challenging [37]. As an alternative to the ICC-based clustering measure in multilevel logistic regression, median odds ratio (MOR) was proposed, which expresses the between-cluster variance on the odds ratio (OR) scale [39]. The values of MOR = 1 indicate the absence of between-cluster heterogeneity, and MOR> 1 indicates there is considerable between-cluster heterogeneity [39].

All analyses were weighted using CCHS’s prescribed weight variable [28, 40], accounting for the complex survey design to ensure the generalizability of the findings to all Canadian populations. SPSS version 26 and STATA version 12 were used to carry out the analysis.

## Results

The present analysis was conducted based on a total of 63,815 adult respondents (aged 18 and above) who were working during the 12 months prior to the survey. Of those, 18.7% were obese based on their self-reported BMI. Table 1 displays the characteristics of the study participants (weighted). Overall, nearly one-half of the participants were males. About 71% of the study participants were white Canadians, and 72.3% of the respondents were non-immigrants (Canadian-born). Close to half of the respondents were married, 69.0% reported having a post-secondary education. The largest proportion of participants was age 18–34 years (35.5%), followed by those in the 35–49-year age group (31.4%). Most of the respondents reported an average annual income of $80,000 or more. The proportions of employee and self-employed were 75.8 and 13.7%, respectively. About 45% of the study participants were categorized as physically active and 16.0% sedentary. More than two-thirds of the respondents reported that they experienced moderate to severe work-related and life stress. Close to 18.0% were either occasional or daily smokers, and almost 70.0% of the respondents were regular drinkers (see Table 1).

**Table 1:** Percentage distribution of participants’ characteristics, CCHS 2017–18, Canada (*n* = 63,815)

Characteristics	Weighted %	Missing (%)
**Sex**		0.0
Male	52.7	
Female	47.3	
**Ethnicity**		0.0
White	70.8	
Aboriginal	3.7	
Visible minority	25.5	
**Age**		0.0
18–34	35.5	
35–49	31.4	
50–64	28.1	
65 and above	5.0	
**Marital status**		0.2
Married	47.9	
Common-law	15.2	
Widowed/Divorced/Separated	8.3	
Single	28.4	
**Educational level**		1.4
Less than secondary school graduation	6.2	
Secondary school graduation	23.5	
Post-secondary certificate diploma/university	68.9	
**Immigration Status**		1.6
Landed immigrant	26.1	
Non-immigrant (Canadian born)	72.3	
**Household food security**		1.9
Food secured	90.8	
Moderately food insecure	5.2	
Severely food insecure	2.1	
**Household income**		0.1
No income or less than $20,000	4.0	
$20,000 to $39,999	9.2	
$40,000 to $59,999	12.7	
$60,000 to $79,999	12.7	
$80,000 or more	61.3	
**Employment type**		10.5
Employee	75.8	
Self-employed	13.7	
**Number of hours of work/per week** Median (IQR)	40 (10)	11.3
**Physical activity**		2.4
Active	44.6	
Moderately active	17.3	
Mild active	20.1	
Sedentary	15.7	
**Work stress level**		2.2
Not at all stressful	9.5	
Not very stressful	19.9	
A bit stressful	41.3	
Quite a bit stressful	22.3	
Extremely stressful	4.7	
**Life stress**		0.3
Not at all stressful	8.7	
Not very stressful	21.5	
A bit stressful	44.7	
Quite a bit stressful	21.4	
Extremely stressful	3.4	
**Smoking cigarettes**		0.1
Not at all	82.0	
Occasionally	5.7	
Daily	12.2	
**Alcohol consumption**		0.4
Regular drinker	69.0	
Occasional drinker	15.3	
Did not drink in the last twelve months	15.3	
**Obesity status**		
Not obese	76.8	4.5
Obese	18.7	

Table 2 presents the percentage distribution of the risk factors stratified by the obesity status based on the bivariate mixed-effect logistic regression analysis. It is noted that all the variables had an association with obesity. Thus, all the variables are further entered into the multivariable mixed-effect logistic regression to examine the net effects of exposure to work and life stress controlling for all other variables in the model (See Table 3).

**Table 2:** Percentage distribution and unadjusted odds ratios with 95% confidence intervals for the association of sociodemographic and lifestyle factors with obesity, CCHS, 2017–2018, Canada

	Obesity		Unadjusted	
	No(%)	Yes (%)	OR (95% CI)	***p***-values
**Sociodemographic variables**				
**Sex**				
Male	79.2	20.8	1	< 0.001
Female	81.9	18.1	0.840 (0.808–0.874)	< 0.001
**Ethnicity**				
White	78.3	21.7	1	
Aboriginal	70.6	29.4	1.401 (1.272–1.543)	< 0.001
Visible minority	87.9	12.1	0.593 (0.562–0.627)	< 0.001
**Age**				
18–34	86.0	14.0	1	
35–49	78.0	22.0	1.725 (1.642–1.812)	< 0.001
50–64	76.7	23.3	1.832 (1.742–1.927)	< 0.001
65 and above	78.4	21.6	1.652 (1.502–1.816)	< 0.001
**Marital status**				
Married	78.8	21.2	1	
Common-law	79.4	20.6	0.931 (0.879–0.986)	0.015
Widowed/Divorced/Separated	77.3	22.7	1.084 (1.008–1.166)	< 0.001
Single	84.7	15.3	0.675 (0.643–0.709)	< 0.001
**Educational level**				
Less than secondary school graduation	73.6	26.4	1	
Secondary school graduation	79.2	20.8	0.750 (0.691–0.815)	< 0.001
Post-secondary certificate diploma/university	81.5	18.5	0.684 (0.634–0.739)	< 0.001
**Immigration Status**				
Landed immigrant	87.1	12.9	1	
Non-immigrant (Canadian born)	77.9	22.1	1.582 (1.500–1.667)	< 0.001
**Household food security**				
Food secure	80.8	19.2	1	
Moderately food insecure	75.7	24.3	1.361 (1.253–1.479)	< 0.001
Severely food insecure	70.6	29.4	1.855 (1.642–2.094)	< 0.001
**Household income**				
No income or less than $20,000	82.3	17.7	1	
$20,000 to $39,999	78.3	21.7	1.243 (1.103–1.402)	< 0.001
$40,000 to $59,999	80.0	20.0	1.086 (0.967–1.219)	0.165
$60,000 to $79,999	80.1	19.9	1.058 (0.943–1.188)	0.337
$80,000 or more	80.8	19.2	1.035 (0.932–1.150)	0.516
**Employment type**				
Employee	80.2	19.8	1	
Self-employed	80.8	19.2	0.989 (0.986–0.992)	< 0.001
**Number of hours of work/per week**			1.006 (1.004–1.007)	< 0.001
**Lifestyle variables**				
**Physical activity**				
Active	82.6	17.4	1	
Moderately active	79.9	20.1	1.232 (1.166–1.302)	< 0.001
Mild active	78.7	21.3	1.288 (1.223–1.357)	< 0.001
Sedentary	77.2	22.8	1.411 (1.334–1.492)	< 0.001
**Work stress**				
Not at all stressful	81.1	18.9	1	
Not very stressful	82.5	17.5	0.923 (0.853–0.998)	0.045
A bit stressful	80.6	19.4	1.051 (0.979–1.128)	0.170
Quite a bit stressful	79.4	20.6	1.140 (1.057–1.230)	< 0.001
Extremely stressful	74.7	25.3	1.520 (1.371–1.686)	< 0.001
**Life stress**				
Not at all stressful	81.8	18.2	1	
Not very stressful	82.0	18.0	0.977 (0.902–1.060)	0.581
A bit stressful	80.4	19.6	1.095 (1.016–1.179)	0.017
Quite a bit stressful	78.9	21.1	1.235 (1.140–1.337)	< 0.001
Extremely stressful	76.4	23.6	1.419 (1.258–1.602)	< 0.001
**Smoking cigarettes**				
Not at all	80.2	19.8		
Occasionally	85.9	14.1	0.647 (0.589–0.712)	< 0.001
Daily	79.7	20.3	0.966 (0.910–1.025)	0.251
**Alcohol consumption**				
Did not drink in the last twelve months	80.6	19.4		
Occasional drinker	75.6	24.4	1.272 (1.187–1.363)	< 0.001
Regular drinker	81.5	18.5	0.900 (0.851–0.952)	< 0.001

**Table 3:** Adjusted odds ratios with 95% confidence intervals for the association of sociodemographic and lifestyle factors with obesity, CCSH, 2017–2018, Canada. (*n* = 63,815)

Sociodemographic Characteristics	AOR (95% CI)	***p***-values
**Intercept**	0.191 (0.154–0.238)	0.000
**Sex**		
Male	1	< 0.001
Female	0.780 (0.745–0.817)	< 0.001
**Ethnicity**		
White	1	
Aboriginal	1.438 (1.287–1.607)	< 0.001
Visible minority	0.657 (0.607–0.712)	< 0.001
**Age**		
18–34	1	
35–49	1.571 (1.478–1.668)	< 0.001
50–64	1.614 (1.513–1.722)	< 0.001
65 and above	1.574 (1.393–1.778)	< 0.001
**Marital status**		
Married	1	
Common-law	0.938 (0.878–1.002)	0.057
Widowed/Divorced/Separated	0.915 (0.841–0.995)	0.039
Single	0.800 (0.750–0.854)	< 0.001
**Educational level**		
Less than secondary school graduation	1	
Secondary school graduation	0.872 (0.793–0.960)	0.005
Post-secondary certificate diploma/university	0.748 (0.683–0.819)	< 0.001
**Immigration Status**		
Landed immigrant	1	
Non-immigrant (Canadian born)	1.462 (1.357–1.575)	< 0.001
**Household food security**		
Food secure	1	
Moderately food insecure	1.386 (1.256–1.528)	< 0.001
Severely food insecure	1.685 (1.447–1.963)	< 0.001
**Household income**		
No income or less than $20,000	1	
$20,000 to $39,999	1.156 (0.997–1.340)	0.055
$40,000 to $59,999	0.921 (0.798–1.063)	0.262
$60,000 to $79,999	0.893 (0.774–1.031)	0.123
$80,000 or more	0.829 (0.724–0.950)	0.007
**Employment type**		
Employee		
Self-employed	0.843 (0.792–0.899)	< 0.001
**Number of hours of work / per week**	1.003 (1.001–1.005)	< 0.001
**Lifestyle variables**		
**Physical activity**		
Active	1	
Moderately active	1.250 (1.176–1.328)	< 0.001
Mild active	1.300 (1.227–1.378)	< 0.001
Sedentary	1.385 (1.299–1.476)	< 0.001
**Work stress**		
Not at all stressful	1	
Not very stressful	0.882 (0.803–0.970)	0.010
A bit stressful	0.950 (0.868–1.040)	0.268
Quite a bit stressful	1.003 (0.907–1.109)	0.958
Extremely stressful	1.432 (1.248–1.644)	< 0.001
**Life stress**		
Not at all stressful	1	
Not very stressful	0.999 (0.906–1.102)	0.984
A bit stressful	1.050 (0.956–1.153)	0.309
Quite a bit stressful	1.077 (0.970–1.196)	0.163
Extremely stressful	0.994 (0.849–1.165)	0.945
**Smoking cigarettes**		
Not at all	1	
Occasionally	0.698 (0.628–0.777)	< 0.001
Daily	0.771 (0.719–0.827)	< 0.001
**Alcohol consumption**		
Did not drink in the last twelve months	1	
Occasional drinker	1.219 (1.125–1.320)	< 0.001
Regular drinker	0.813 (0.759–0.870)	< 0.001
**Estimated variance parameter of the random effect term**	0.081 (0.056–0.116)	< 0.001

Intraclass correlation coefficient (ICC) = 0.024

Median Odds Ratio (MOR) = 1.31

The results of the adjusted mixed-effect logistic regression model for the effects of work and life stress on obesity are presented in Table 3. Prior to running the multivariable model, multicollinearity among the factors was examined, and none of them had significant collinearity (VIF < 2.5), as shown in Table S1 in the Supplementary Materials. The variance component of the random effect term of the multivariable model is estimated as 0.081 (95% CI: 0.056–0.116) (*p* < 0.001), which suggests a moderate correlation of individuals within the health region in the residuals that are unexplained by the fixed effect covariates. The ICC is estimated as 0.024, and the MOR is 1.31, indicating moderate level heterogeneity between the health regions (Table 3).

The odds of obesity decreased by 22% for females (AOR = 0.780; 95% CI: 0.745–0.817) compared to males. Aboriginal respondents were 1.438 times (95% CI: 1.287–1.607) more likely to be obese, and the odds decreased by 34.3% for visible minorities compared to white Canadians (AOR = 0.657; 95% CI: 0.607–0.712). Similarly, there was a lower likelihood of experiencing obesity among those living in the highest income categories (80,000 dollars or more) than those in the lowest income category. Respondents residing in households with moderate and severe food insecurity were 1.386 (95% CI: 1.256–1.528) and 1.685 (95% CI: 1.447–1.963) times more likely to experience obesity. For every one-hour increment in the number of hours of work per week, the odds of obesity increase by 1.003 times (95% CI:1.001–1.005). In other words, for every 10 h increment in the number of hours of work per week, the odds of obesity increase by (1.003)^10^ = 1.03 times.

All the lifestyle variables are strongly associated with obesity. The odds of the outcome were significantly higher among those who reported they had moderate to severe work-related stress. For instance, the odds of obesity were 1.432 times (95% CI: 1.248–1.644) among those who reported extreme work-related stress, compared to those who had no work-related stress. Relative to physically active participants, those having sedentary lifestyles had a higher chance of being obese, and the AOR increases as the level of physical activity decreases. The likelihood of obesity was found to be lower among occasional and daily smokers. Similarly, alcohol intake was inversely associated with the risk of obesity. Further analysis of the data showed no meaningful two-way interactions of the key explanatory variables.

## Discussion

This study reveals important associations between stress and obesity among the working adult Canadian population based on a nationally representative data from Canada (2017–2018). The study found that more than two-thirds of the respondents reported that they experienced moderate to severe work-related and life stress. Previous studies in Canada reported that over a quarter of working Canadians report daily high stress [41] and about two-thirds (65%) reported work situations as the main source of their stress [42]. The results of adjusted mixed-effect logistic regression showed that the likelihood of obesity was higher among those with experiences of severe work stress, those with sedentary lifestyles, among non-immigrants, older people, aboriginals, respondents working as an employee (compared to self-employed), and those experiencing severe to moderate food insecurity. The odds were lower for females, visible minorities, and respondents with better education and higher household income.

The present study has found a significant association between work stress variable and the likelihood of obesity among the adult population of Canada. Previous studies conducted in Canada have reached a similar conclusion. For instance, Chen and Qian (2012) based on Canadian national data of 2006–2007 reported that people who had higher stress had a higher likelihood of becoming obese [26]. Similarly, another study conducted on Canadian adults [27], reported that stress significantly increased the Body Mass Index (BMI) of adults. However, those studies had a small number of control variables based on conventional logistic regression models. Though understanding the mechanisms through which stress impacts obesity is somewhat difficult, evidence suggests that chronic stress is a function of biological and behavioral effects of stress [21]. A plausible biological explanation is that stress increases a body hormone called ‘cortisol’ [22–24], leading to craving unhealthy food, consuming food with a high glycemic index, overeating, and a reduced amount of sleep [22, 24, 43]. Further, it is reported that stress not only impacts the ability to maintain a healthy weight but also makes it more difficult to lose weight [22]. Although stress is usually considered a cause of obesity, a reverse association could also be possible [44].

Some evidence suggests that the association between work stress and obesity could also depend on the nature of work, the amount of time individuals spend on the work and the level of social support at the workplace. In the present study, self-employed individuals were less likely to become obese compared to employees. Similarly, the number of hours of work and obesity are positively associated. A study conducted in Canada reported that men working longer hours (more than 40 per week) had an increased chance of being obese compared to regular full-time workers [17]. In the same study, a greater proportion of shift workers (both men and women) were obese compared with regular-schedule workers [17]. It is also important to note that work stress could be combined with low social support at the workplace and poor coping mechanisms. In other words, obese workers perceived not only high levels of job strain, but also insufficiency of an important buffer against work stress [17]. High psychological workload, together with a lack of proper social support at work, may act as a causal factor for obesity [17].

Interestingly, our findings show that regular drinking of alcohol and cigarette smoking results in decreased odds of obesity. Several studies have conflicting conclusions [45, 46], but recent studies documented that the two are inversely associated. For instance, in a community-based study in Saudi Arabia, the prevalence of obesity was lower among current smokers compared to nonsmokers and ex smokers [45]. Patel and colleagues showed that current smokers had decreased odds of being overweight or obese compared to normal-weight non-smokers, among both African American and Caucasian women [46]. Several other studies have also reported that smoking was associated with lower weights and BMI [47–49]. One plausible explanation for such association could be nicotine’s ability to increase energy expenditure and could reduce appetite until the nicotine-induced set-point is achieved [50]. Previous studies regarding the association between alcohol consumption and obesity are mixed. For instance, Rohrer and colleagues (2005) concluded that people who consumed alcohol three or more days per month had lower odds of being obese compared to non-drinkers [51]. On the contrary, a Korean study reported high alcohol consumption significantly associated with higher odds of obesity, even after adjustment is made for clinical factors [52]. Some studies explain such discrepancies as differences in drinking patterns behaviors, including the culture of drinking, which varies across different populations [53, 54].

The level of physical activity, which is commonly used as a proxy for a sedentary lifestyle, has shown a significant association with obesity. The finding agrees with previous studies conducted in Canada [1, 15, 18]. Once a person becomes obese, there is a high likelihood that obesity itself discourages physical activity [20] and leads to likely engagement in more sedentary jobs [55]. One of the well-established pathways for physical activity is that it is instrumental in reducing excess fat and, hence, significantly reduces morbidities and mortality [17].

Our results also indicated that obesity was associated with some socio-demographic factors, which were included in this study as control variables. The significant background variables are age, sex, household income, household food security status, educational status, ethnicity, immigration status and marital status. As expected, obesity tends to increase with age, with a higher prevalence at older ages. It is well established from previous studies that the incidence of obesity increases with age. Studies conducted in Canada reached a similar conclusion [1, 15]. One plausible reason for this could be related to numerous age-related changes in the physiological state of individuals [56]. Our findings also indicated lower odds of obesity among single, widowed, divorced and those in common marriage compared to married women. While the pathways for such association could be complex to locate, one plausible reason could be that never-married people tend to put more value on their body image when they are young [17]. The results of this study also showed aboriginal respondents had higher odds of obesity, while the odds significantly decreased for visible minorities compared to white respondents. This finding aligns with those of the previous studies concluding that aboriginals in North America (US) were more likely to encounter poor health outcomes, including morbidities and mortality events [57]. Interestingly, we found an inverse association between education and obesity in the present study. Most existing evidence suggests that behind socioeconomic gradients in health are a higher prevalence of risky health behaviors among the poor, which are posited as the actual cause of socioeconomic gradients in health [17, 58]. The finding is consistent with the overall understanding that better education promotes health behavior, as people with better education may better understand and adhere to prevention practices [1, 15, 59]. We found a strong inverse association between household income and the likelihood of obesity, indicating that the odds of obesity decreased for the highest income group. A related variable, household food security, was inversely associated with the outcome variable of interest. The odds of obesity have become higher for households with severe and moderate household food insecurity. It is obvious that individuals living in severe food insecure households tend to make no choice of healthy foods. It should be noted that the mechanisms by which social-economic status affects health outcomes likely involves multiple interacting material, behavioral, and psychosocial factors occurring throughout the life course [59].

### Strength and limitations

The findings of this study were based on nationally representative survey data from Canada. Thus, the findings can be generalized to the Canadian population. Given the importance of understanding the mechanisms of obesity in its prevention, the results will contribute to the planning, monitoring, and evaluation of health outcomes, especially obesity. It may also serve as a reference point for future researchers to analyze more distal factors in-depth. However, this study is not without limitations. As the data were generated through a cross-sectional survey, causal inferences between stress variables and obesity were not possible. The self-report nature of obesity might also impact the accuracy of the measurement of the outcome due to possible inaccuracy and omission of information by participants. The report from Statistics Canada showed that the prevalence of obesity might be underestimated with self-reported data [60]. A long recall period for stress, as well as simple and direct questions on self-reported stress, might bias the responses. Finally, this study did not consider some lifestyle variables (such as diet, medications, and chronic conditions) due to a large number of missing values in those variables.

## Conclusion

The study found that 18.7% of the working adult population was obese. It was noted that work stress and other socioeconomic variables significantly determine the risk of obesity. Given the ever-changing work and life situations and the prospects of increased stress, the findings call for public health attention. Improving social support systems at the workplace and individual/group counseling on managing stressors and drivers could make significant program impacts. The findings call for policymakers and health administrators to devise evidence-informed strategies at the health system and macro-level directed at managing obesity in working adults by paying more attention to combating stress. Therefore, intervention efforts should allocate more resources on Behavioral Change Communication (BCC), focusing on the home and work environment such as leisure, stress management and coping, and other targeted public health education. Finally, more rigorous longitudinal studies should be done to document changes in behaviors, and interactions of stress variables with other key variables.

## Supplementary Information

**Additional file 1: Table S1.** Variance Inflation Factor (VIF) for examining multicollinearity among the explanatory variables.

## Data Availability

Access to the data is available to bona fide researchers through institutions participating in Statistics Canada Data Liberation Initiative (DLI) including university libraries throughout Canada – see https://www.statcan.gc.ca/eng/dli/dli.

## Abbreviations

CCHS: Canadian Community and Health Survey
CI: Confidence Interval
OR: Odds Ratio
SDGs: Sustainable Development Goals
SEM: Social Ecological Model
WHO: World Health Organization
