# Supplementary material for: Examining the association between work stress, life stress and obesity among working adult population in Canada: findings from a nationally representative data
Source: Arch Public Health. 2022 Mar 29;80:97. doi: 10.1186/s13690-022-00865-8 (PMC8966340; doi:10.1186/s13690-022-00865-8)
Supplement: Supplementary file 1 — Additional file 1: Table S1. Variance Inflation Factor (VIF) for examining multicollinearity among the explanatory variables. [file 13690_2022_865_MOESM1_ESM.docx]

**Table S1:** Variance Inflation Factor (VIF) for examining multicollinearity among the explanatory variables.

|  | **Collinearity Statistics** | |
| --- | --- | --- |
| Variables | Tolerance | VIF |
| Sex | .904 | 1.106 |
| Marital status | .769 | 1.300 |
| Highest level of education | .916 | 1.092 |
| Perceived life stress | .670 | 1.493 |
| Perceived stress at work | .647 | 1.545 |
| Type of smoker | .931 | 1.074 |
| Physically active based on WHO guidelines | .940 | 1.063 |
| Immigration status | .561 | 1.781 |
| Total household income - all sources | .834 | 1.199 |
| Household food security status | .925 | 1.081 |
| Employee or self-employed | .934 | 1.071 |
| Racial status of respondents | .553 | 1.808 |
| Age group | .806 | 1.241 |
| Type of drinker - 12 months | .860 | 1.163 |
| Total usual hours worked per week | .870 | 1.149 |
